# Trends of Antibacterial Resistance at the National Reference Laboratory in Cameroon: Comparison of the Situation between 2010 and 2017

**DOI:** 10.1155/2021/9957112

**Published:** 2021-05-22

**Authors:** M. Massongo, L. Ngando, E. W. Pefura Yone, Ariane NZouankeu, W. Mbanzouen, M. C. Fonkoua, A. Ngandjio, J. Tchatchueng, D. Barger, M. C. Tejiokem

**Affiliations:** ^1^Faculty of Medicine and Biomedical Sciences, University of Yaoundé 1, Yaoundé, Cameroon; ^2^Jamot Hospital, Yaoundé, Cameroon; ^3^Bacteriology Unit, Cameroon Pasteur Centre, Member of Institut Pasteur International Network, Yaoundé, Cameroon; ^4^Epidemiology and Public Health Unit, Cameroon Pasteur Centre, Member of Institut Pasteur International Network, Yaoundé, Cameroon; ^5^Bordeaux School of Public Health (ISPED), University of Bordeaux, Inserm 1219 Bordeaux Population Health, Bordeaux, France

## Abstract

**Introduction:**

Antimicrobial resistance represents a growing public health threat. One of the World Health Organization's strategic objectives is “strengthening knowledge through surveillance and research.” Sub-Saharan African countries are still far from achieving this objective. We aimed to estimate and compare the prevalence of antibacterial resistance in 2010 and 2017 in Cameroon.

**Methods:**

We conducted a retrospective study on all clinical specimens cultured in Centre Pasteur du Cameroun (CPC) in 2010 and 2017. Data were extracted from the CPC's laboratory data information system software and then managed and analyzed using R. Bacterial resistance rates were calculated in each year and compared using chi-square or Fisher's tests, and relative changes were calculated. Outcomes included acquired resistance (AR), WHO priority resistant pathogens, some specific resistances of clinical interest, and resistance patterns (multi, extensively, and pan drug resistances) for five selected pathogens.

**Results:**

A total of 10,218 isolates were analyzed. The overall AR rate was 96.0% (95% CI: 95.4–96.6). Most of WHO priority bacterial resistance rates increased from 2010 to 2017. The most marked increases expressed as relative changes concerned imipenem-resistant *Acinetobacter* (6.2% vs. 21.6%, +248.4%, *p* = 0.02), imipenem-resistant *Pseudomonas aeruginosa* (13.5% vs. 23.5%, +74.1%, *p* < 0.01), 3rd generation-resistant Enterobacteriaceae (23.8% vs. 40.4%, +65.8%, *p* < 10^−15^), methicillin-resistant *Staphylococcus aureus* (27.3% vs. 46.0%, +68.6%, *p* < 0.002), fluoroquinolone-resistant *Salmonella* (3.9% vs. 9.5%, +142.9%, *p* = 0.03), and fluoroquinolone-resistant Enterobacteriaceae (32.6% vs. 54.0%, +65.8%, *p* < 10^−15^). For selected pathogens, global multidrug resistance was high in 2010 and 2017 (74.9% vs. 78.0% +4.1%, *p* = 0.01), intensively drug resistance rate was 5.8% (7.0% vs. 4.7%; *p* = 0.07), and no pan drug resistance has been identified.

**Conclusion:**

Bacterial resistance to antibiotics of clinical relevance in Cameroon was high and appeared to increase between 2010 and 2017. There is a need for regular surveillance of antibacterial resistance to inform public health strategies and empirically inform prescription practices.

## 1. Background

Antimicrobial resistance (AMR) has increased within the past years, and according to the World Health Organization (WHO), this is a growing threat for public health worldwide that calls for urgent and global measures [1]. Although AMR is a natural phenomenon, human factors have a significant impact on its spread. Such factors can be behavioral (inadequate use of antimicrobials, lack of hygiene), structural (weakness in laboratory supply), or managerial (lack of surveillance, inadequate or lacking control programs, poor regulation) [2]. Inadequate use of antimicrobials is one of the major AMR suppliers. This includes antibiotic adds in drinking water and daily feed of food-producing animals [3], as well as the intensive use of growth promoters that can encourage the growth of selected resistant bacteria [4]. The overuse, subtherapeutic, or other misuse of antibiotics in animals has shown a correlation with the occurrence of resistant bacteria in humans. These resistant bacteria range from *Escherichia coli* to *Mycobacterium bovis* [5–8]. Some factors are more likely to occur in low income countries, such as limited access to a right diagnosis or illicit drug to sell and use, among others [9]. Unregulated antimicrobial use and nonestimation of annual usage are also frequent in developing countries, as shown by Rahman in Pakistan [10].

Facing this growing threat, the WHO published in 2014 a global report on antimicrobial resistance surveillance [11]. Based on national, regional, and global data, this report revealed the sanitary and economic impact of AMR, which included among others: a twofold increase of all-cause mortality rate, an increased risk of infection after admission in intensive care unit, an increased hospitalization stay, an increased frequency of septic shock, and a significant increase of management cost. For many sub-Saharan African (SSA) countries including Cameroon, the expressions “No information obtained for this report” or “No national data available” occured in this first report. At that time, studies on AMR in Cameroon were scarce and old. They were limited in terms of geographical areas and pathogens. However, they showed an increasing AMR rate between the 1990s and the late 2000s [12–15]. One year later, WHO published a country situation analysis of the response to AMR [2], based on a 2-year survey with the national sanitary authorities of state members. Only 8 SSA countries (Africa WHO region) out of 47 (17.0%) provided data for this report, while 127/147 (85.0%) responded for the other WHO regions. African countries showed a lack of national AMR policies, lack of sensitization programs, and inappropriate use of antimicrobials, among others. There was no data from Cameroon in this second report.

After those 2 baseline documents, WHO published in 2016 its Global Action Plan on antimicrobial resistance, based on 5 strategic objectives. The objective number 2 was “to strengthen the knowledge and evidence base through surveillance and research” [16]. We aimed to provide data on the recent evolution of AMR in Cameroon, pursuing a dual purpose: public health (contributing to the objective number 2 of WHO's Global Action Plan) and clinical (possible implications on antibiotics protocols).

## 2. Materials and Methods

### 2.1. Study Design and Settings

We performed a retrospective study from April 4 to June 1, 2018, in Centre Pasteur du Cameroun (CPC), Yaoundé, the national reference laboratory in biology and public health, and Member of the Pasteur Institute International Network. As a nationwide reference laboratory, CPC analyzes specimen coming from Yaoundé and other regions of the country. We analyzed bacteriological routine data, collected during two distinct one-year periods (2010 and 2017), and stored in the laboratory data information system (GLIMS). We initially aimed to perform an up-to-date ten-year comparison (2007–2017) of the various AMR features targeted, 2017 being the more recent year with completed data. Unfortunately, GLIMS was put in place in July 2009, making 2010 the first year with comprehensive stored data.

### 2.2. Study Population and Sampling

**Figure 1:**
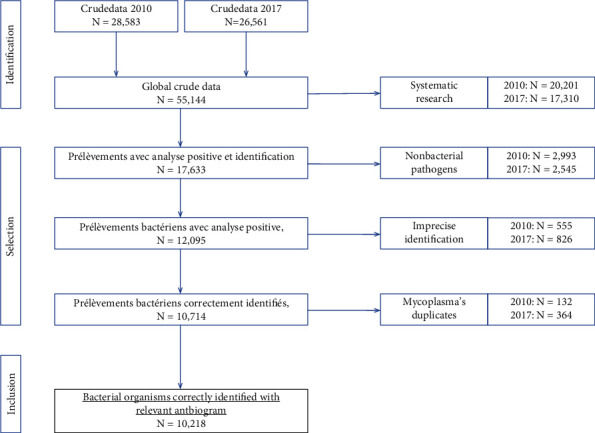
Flow chart of isolate inclusion in the study on antibacterial resistance, Centre Pasteur du Cameroon, 2010 and 2017, Yaoundé, Cameroon.

All bacterial analyses with a valid susceptibility test conducted in CPC in 2010 and 2017 were considered for this study, regardless of their collection site or source of specimens (blood, sputum, urine, stool, pus, etc.). The following were excluded from analysis: samples with isolates from nonpathological swabs (including systematic research with no clinical interest), fungal specimen, or duplicates, especially for the Mycoplasma family, since both *Mycoplasma hominis* and *Ureaplasma urealyticum* were systematically reported when one of them was identified (Figure 1).

### 2.3. Biological Analysis: Procedures and Interpretation

Biological samples had been collected at or transferred to CPC during the enrollment period. For each sample, the name, age, and gender were used to label the corresponding samples and create ID codes for each analysis. Samples were then brought to the bacteriology lab for analysis. Most of the specimen were examined using light microscopy after Gram staining or other appropriate method to assess the presence of pathogens. Blood specimens were incubated at 37°C in BactALERT 3D Automaton (Biomerieux) and examined daily for evidence of bacterial growth. When needed, specimens were cultured according to French microbiology guidelines [17, 18]. Bacteria identification was done using Vitek 2 Compact (Biomerieux) automaton and API kits. The antibiotics susceptibility tests were performed using standard methods (disk, e-test, liquid medium for Vitek 2 from January 2014). Reference documents for these tests were the CASFM (Comité Antibiogramme de la Société Française de Microbiologie) 2010 and 2016 [19, 20]. Results were stored in GLIMS using generated ID codes in tables including patient identification (name, age, gender), sample type or source, microorganism, and susceptibility profile. The later was given through 3 modalities: susceptible (S), intermediate (I), or resistant (R).

### 2.4. Data Management and Analysis

From GLIMS, we extracted all bacteriology analysis stored in 2010 and 2017 into Excel® tables without patients' name. The crude global database underwent the abovementioned filters (Figure 1). The resulting table was then uploaded to R version 3.4.3, for data management and analysis. Variables were renamed and harmonized, and new variables were created to fit with our 3 main outcomes:

- *Global Acquired Resistance*. The susceptibility profile was processed to obtain a binary variable (absence or presence of acquired resistance). There was no acquired resistance (wild strain) if a given bacterium was still fully susceptible (S) to all antibiotics (ATB) naturally active in vitro. There was acquired resistance (resistant strain) if the pathogen had been found intermediate (I) or resistant (R) for at least one of these ATBs
- *Specific Resistance*. This informed susceptibility profile of the bacterium regarding a given ATB and permitted identification of WHO's antibiotic-resistant “priority pathogens.” These are 12 families of bacteria that pose the greatest threat to human health, divided into three categories according to the urgency of the need for new antibiotics: critical (priority 1), high (priority 2), and medium (priority 3) [21]
- *Resistance Pattern*. It is applied to *Enterobacteriaceae*, *P aeruginosa*, *A baumannii*, *Staphylococcus aureus*, and *Enterococcus spp*, which are frequently involved in healthcare-associated infections. For these bacteria, 5 resistance levels were defined by an international expert proposal from the Centre for Disease Control (CDC) and the European Centre for Disease Control (ECDC): wild, acquired resistant (AR), multidrug-resistant (MDR), extensively drug-resistant (XDR), and pan drug-resistant (PDR) bacteria [22]. Wild and resistant bacteria were defined as stated in the variable “susceptibility profile.” MDR was defined as acquired nonsusceptibility to at least one agent in three or more antimicrobial categories. XDR was identified when a bacterial isolate remained susceptible to only one or two ATB categories. PDR was defined as nonsusceptibility to all agents in all antimicrobial categories [22]

For each of these outcomes, the corresponding AMR indicators for a given bacterium or group of bacteria were estimated in 2010 and 2017, using the following formula:

(1) $$\begin{array}{c} \text{Prevalence}=\frac{\text{Number of isolates presenting the given resistance }}{\text{Total number of isolates concerned}}\times 100. \end{array}$$

A relative change (RC) from 2010 to 2017 was then calculated using the following formula:

(2) $$\begin{array}{c} \text{RC}=\frac{\text{Prevalence in }2017-\text{Prevalence in }2010\;}{\text{Prevalence in }2010}\times 100. \end{array}$$

The global prevalence of antibacterial resistance was presented with its 95% confidence interval. The specific AMR rates were given as numbers and percentages. Between-period comparisons were performed using Chi square or Fisher tests. A *p* value <0.05 was considered as significant.

### 2.5. Ethical Statements

This work was approved by CPC's administrative board. Since data had been collected routinely prior to the study and not for a research purpose and personal contact details were not available, individual consents could not be obtained from participants. Data were analyzed anonymously. The study induced no risk for patients or CPC staff.

## 3. Results

### 3.1. Study Population, Specimens, and Bacterial Isolates

A total of 55,144 analysis were extracted; 48.2% of them were realized from 2017. Analyses resulting from systematic demands or screening purpose (such as beta hemolytic streptococci in throat, Escherichia, Campylobacter, and other entero-pathogens in stool, Candida in stool and vaginal fluid, nasal or throat staphylococci, and vaginal streptococcus B) were first excluded, since they did not reflect a clinical infectious situation, and most of them had negative results (which were systematically reported and stored). These 37,511 analyses accounted for 2/3 of the total. Nonbacterial isolates (mainly fungi) were then excluded, followed by bacterial one for which the identification was imprecise or not completed (such as “Gram-negative bacillus,” “Gram-positive bacteria,” and “Other staphylococcus”). Lastly, duplicates of Mycoplasmas (*Mycoplasma hominis* and *Ureaplasma urealyticum*) were eliminated. These duplicates appeared for some isolates and consisted in a second line for the same patient and the same specimen but without any susceptibility test. This selection process led to a final sample of 10,218 isolates (Figure 1).

These isolates originated from 7,314 patients, 3,394 (46.4%) enrolled in 2010, and 3,920 (53.6%) in 2017. Their mean age ± standard deviation was 30.2 ± 21.0 years, nearly 75% of them were adults, and 62.6% were woman. There were 8,985 distinct specimens, from 23 different collection sites. The collection sites were dominated by female genital (32.7%) and urinary (28.7%) ones. These samples were gathered into 4 groups, according to their origin and clinical relevance. Comparison between 2010 and 2017 according to patients' gender and age as well as collection sites is shown in Table 1.

A total of 71 bacteria species were identified and grouped into 14 categories, according to morphological and susceptibility criteria (Table 2). Genital Mycoplasma accounted for one-third of the total. Apart from those genital tract commensals, *Escherichia coli* (17.0%), *Klebsiella pneumoniae* (8.2%), and *Staphylococcus aureus* (5.0%) were the 3 more frequent bacteria identified in this study.

### 3.2. Global Acquired Resistance

Of the 10,218 bacterial isolates, 5,405 (52.8%) underwent a proper acquired resistance estimation. The other were excluded mainly due to missing or discordant data in the susceptibility tests (45.9%) and in a few cases for insignificant number (1.3%).

Among these 5,405 isolates, 5,189 were resistant to at least one antibiotic usually active in vitro, giving an overall prevalence (95% CI) of acquired resistance in this bacterial population of 96.0% (95.4–96.6)%. This prevalence increased from 2010 to 2017 (92.8% to 99.2%, RC = +6.9%, *p* < 0.0001). Four bacterial groups, accounting for 75.3% of the total, had a 100% acquired resistance rate in 2010 and 2017. These were Enterobacteriaceae, other fermenting GNB, Enterococci, and Anaerobic (Table 3). In addition, *Streptococcus pneumoniae* (56 isolates), *Pseudomonas aeruginosa* (195 isolates), and *Acinetobacter spp* (150 isolates) also had a 100% resistance rate at the two data collecting periods.

### 3.3. Specific Resistances

Globally, an increase in WHO priority resistances was observed between 2010 and 2017, with relative changes ranging from +32.4% (Ampicillin-R *Haemophilus* influenzae) to +248.4% (Imipenem-R *Acinetobacter baumannii*). Only imipenem-resistant Enterobacteriaceae were less frequent in 2017 than 2010 (RC of -10.8%). For an unknown reason, fluoroquinolones (FQ) susceptibility was not tested for *Neisseria gonorrhoeae* in 2010, but only in 2017. FQ-R *N gonorrhoeae* had the highest rate (80.9%) among the 12 WHO priority pathogens in 2017. Three specific resistances not observed in 2010 were present in 2017: Vancomycin-resistant *Enterococcus spp*, 3rd generation cephalosporin- (3GC-) resistant *Neisseria gonorrhoeae*, and fluoroquinolone-resistant *Shigella* spp. Concerning other pathogens and resistances, Mycoplasma resistance was high and slightly increased for fluoroquinolones, while it decreased for cyclins, between 2010 and 2017. There was a >100% increase in 3GC-resistant *Escherichia coli* (Table 4).

### 3.4. Resistance Patterns

As mentioned in Material and Methods, resistance pattern determination applied to *Enterobacteriaceae*, *P aeruginosa*, *A baumannii*, *Staphylococcus aureus*, and *Enterococcus spp*. Overall, 4,903 (47.9%) strains were eligible. Among these, 4,474 fulfilled the required criteria, on which 3,422 (76.5%) were classified multidrug resistance (MDR). Global resistance patterns and their comparison between 2010 and 2017 are shown in Table 5.

No pan drug resistance (PDR) was identified. The between-period comparison differed from one bacterium to another. MDR increased distinctly for *S. aureus*, while it tended to remain stable for the 4 other pathogens. Conversely, extensively drug resistance (XDR) increased very highly for *A. baumannii* and decreased for the other pathogens, although most of the differences were not statistically significant (Table 6).

## 4. Discussion

More than 10,000 antibiotic susceptibility tests were analyzed in this study. Genital mycoplasmas were the most frequent bacteria, followed by *E. coli*. Swabs were clinically relevant for >90% of them. Almost 100% of strains had an acquired resistance at baseline. All WHO priority resistances highly increased between 2010 and 2017 except Imipenem-R Enterobacteriaceae which slightly decreased. Three resistances not seen in 2010 were present 2017 in this category (Vancomycin-R *Enterococcus spp*, 3GC-R *Neisseria gonorrhoeae*, and Fluoroquinolone-R *Shigella spp*). The prevalence of penicillin nonsusceptible *S. pneumoniae* was low but increased between the 2 periods, while >50% of *H. influenzae* were initially ampicillin-R. FQ-R Enterobacteriaceae were frequent in 2010 and almost doubled 7 years later. Less than 5% of genital mycoplasmas were still fully susceptible to FQ, while the susceptibility to josamycine remained active for nearly all of them. More than 75% of eligible strains were MDR, and no PDR was identified.

Our sample size allowed relevant counts for most of bacteria of clinical interest. This size was higher than most of those we found in other Cameroonian or African studies [12–14, 23, 24]. Age distribution of providing patients (74.5% adults and 21.1% < 5 years) differed from the national one, which shows less adults (53.9%) and more under 5 children (30.5%) [25]. The high frequency of female genital swabs in our sample could partly explain this gap. The predominance of enterobacteria (and especially *E. coli*) in our sample is consistent with the human bacterial pathogen's distribution.

The very high prevalence of AR was predictable. This high prevalence is now well documented and is one of the reasons for global mobilization on AMR. Interestingly, bacteria who scored a 100% AR rate in 2010 and 2017 are among the most frequent human pathogens (Enterobacteriaceae and other GNB). This also makes them the most exposed to antibiotic pressure (including inadequate use), which is thought to be involved in resistance occurrence [26].

In our knowledge, few has been published on class 1 WHO priority resistance (especially Imipenem-R *A baumannii*, Imipenem-R *P aeruginosa*, Imipenem-R Enterobacteriaceae) in Africa. However, recent studies reported imipenem-R *Pseudomonas aeruginosa* (Ipm-R Pa) rate of 11% in Nigeria [27] and 33% in Uganda [28], while we had 23.5% in 2017. An older South African study found a 42% Ipm-R Pa rate in 2007 [29], quite higher than what we got. Comparable disparities were observed for Imp-R *Acinetobacter baumannii*. Such disparities may be related to several factors such as drug policies, carbapenem availability, or financial access. These SSA rates of Imp-R non fermenting GNB (NF GNB) were quite higher than those observed in Western Europe in 2017-2018, which were <10% [30–32]. However, they were still lower than the Iranian 54% [33] and the 66 to 90% range found in Latin America [34]. The rapid increase of Imp-R NF GNB in Cameroon should lead to caution in antibiotic use, since there is no alternative to face highly resistant bacteria in the country.

Carbapenem-R Enterobacteriaceae appeared to have a low and stable frequency in our study. Higher values were found in Morocco [35]. Our 3GC-R Enterobacteriaceae prevalence was lower than those found in earlier Cameroonian studies, around 50% [12–14, 23, 36], probably due to sampling disparities. Since our enrollment was more global (including outpatients and hospitalized ones) than the one in those studies (hospitalized patients only). We found no study on Vancomycin-R enterococcus in Africa, which appeared to be emerging in Cameroon. This may cause serious therapeutic issues. Methicillin-R *Staphylococcus aureus* (MRSA) was more frequent in our study than in other SSA recent studies, where its prevalence ranged from 0% in Zambia to 7% in Zimbabwe [37–39]. This high prevalence may compromise patient management, since the Cameroonian minimum wage, 52.3 euros [40], cannot afford a daily dose of vancomycin (unique anti-MRSA available in Cameroon). Our FQ-R Campylobacter prevalence was also high compared to other SSA series, ranging from 20 to 33% [41, 42]. Meanwhile, FQ-R Salmonella in 2010 close to 4.3% found one year later in Congo [43]. The seven-year increase (+142%) of the latter is easily explained by a very anarchical and overuse of FQ in our country. This overuse could also explain the very high frequency of FQ-R Neisseria gonorrhoeae. Ampicillin-R *H. influenzae* showed very high values, compared to 8.7–46% range found in other countries [35, 44, 45].

The formula used to identify MDR could explain discrepancies with related specific resistances (MRSA and 3GC-R Enterobacteriaceae for example), since it included several antibiotics rather than specific ones. MDR/XDR *Acinetobacter spp* prevalence was consistent with the worldwide survey led by Lob in 2016; it ranged from 47% in Northern America to 93% in Middle East [46]. We found no study from Africa using the same definition of MDR and XDR for other eligible pathogens. A local comparison could therefore not be made.

Our study design did not permit to assess what happened within the 2010–2017 interval. Therefore, real trends could be different from what our results assumed. The retrospective enrollment led to a huge number of excluded specimen and missing data, increasing risk of information bias. Data on providing patients were insufficient, making clinical correlation impossible and population profile nonassessable. However, the accessibility and affordability of CPC allow inclusion of patients from different regions and social categories. Every citizen can realize tests in CPC, and discounts up to 50% are applied for students, children, the elderly, civilian servants, and hospitalized patients from public hospitals. Whole-year enrollment was an advantage since it allowed to overcome potential between-season variations in circulating pathogens. This study succeeded in informing one of the WHO action plan's objectives, especially with the assessment of WHO priority resistances. Using data from a reference laboratory with cutting-edge equipment was a quality guarantee.

Taking into consideration, these AMR's prevalence may help Cameroonian clinicians in their daily practice, given that very few health facilities all around the country can attend a proper bacteriologic test. High prevalence of serious resistances could encourage decision-makers to build programs addressing AMR in the country. They could also be used to sensitize health workers and medical students on the reality of this phenomenon in the country and support training on this important issue.

Clinical data related to identify bacteria could not be reached. Prospective designs should overcome this limitation. The real dynamic of AMR evolution could not be assessed in this study, but trends have been revealed. Repeated similar studies or cohorts would be needed to have a long-term evolution. Extrapolation of these results to the whole country should be cautious, and local or regional studies would be necessary to complete them. Circulating bacteria are not limited to humans and patients; environment and animals must be studied as well.

## 5. Conclusion

In this study designed to assess AMR trends in Cameroon, most of bacteria of clinical interest have shown high resistance rates, which increased between 2010 and 2017, especially WHO priority pathogens. Almost all the strains identified had an acquired resistance. The prevalence of MDR was high, while XDR remained low but increased, and PDR was absent. These results contributed to improve knowledge on AMR in Cameroon and advocate for an urgent need of public health strategies against this threat. There is a need for regular surveillance of antibacterial resistance to keep decision-makers and clinicians informed.

## Figures and Tables

**Table 1:** Collection sites distribution, concerning bacterial analysis in 2010 and 2017, Centre Pasteur du Cameroon, Yaounde. Continuous data are expressed in counts ± standard deviation and categorical data in counts (frequencies in %).

Variable	Modalities	Overall	2010	2017	*p* value
Sex^∗^ *N* = 7,588	Female	4,748 (62.6)	2,274 (63.4)	2,474 (61.9)	0.187
	Male	2,840 (37.4)	1,315 (36.6)	1,525 (38.1)	
	Total	7,588 (100.0)	3,589 (100.0)	3,999 (100.0)	

Age^∗^ *N* = 7,711	Mean	30.2 ± 21.0	29.2 ± 20.0	31.1 ± 22.0	<0.001
	Adult (>18 years)	5,742 (74.5)	2,684 (74.9)	3,058 (74.0)	0.343
	Teen/child (≤18)	1,969 (25.5)	897 (25.1)	1,072 (26.0)	
	Total	7,711 (100.0)	3,581 (100.0)	4,130 (100.0)	

Specimen group^∗∗^ *N* = 8985	Deep	4,350 (48.4)	2,012 (48.1)	2,238 (48.7)	0.280
	Female genital	3,290 (36.6)	1,525 (36.4)	1,765 (36.8)	
	Male genital	711 (7.9)	332 (7.9)	379 (7.9)	
	Superficial	634 (7.1)	319 (7.6)	315 (6.6)	
	Total	8,985 (100.0)	4,188 (100.0)	4,697 (100.0)	

^∗^Sex and age were those informed on each collection site. Given the possibility for a patient to provide more than one sample, there were more samples than patients. ^∗∗^Deep specimen included blood cultures, urine, stool, foreign bodies, soft tissue, broncho-pulmonary, bone-and-joints, pleural effusion, cerebrospinal fluid, intraperitoneal, liver punctures, cerebral, lymph nodes, and gastric liquid. Superficial ones were skin, throat, nasal or wound swab, and any externalized fluid.

**Table 2:** Bacterial groups identified in 2010 and 2017 in Centre Pasteur du Cameroun.

Bacterial group	Isolates 2010 (%)	Isolates 2017 (%)	Over all (%)
Genital Mycoplamas	1,637 (34.8)	1,852 (33.6)	3,489 (34.1)
Group 1 Enterobacteriaceae	1,162 (24.7)	1,057 (19.2)	2,219 (21.7)
Staphylococci	375 (8.0)	669 (12.1)	1,044 (10.2)
Group 2 Enterobacteriaceae	456 (9.7)	469 (8.5)	925 (9.1)
Streptococci	319 (6.8)	321 (5.8)	640 (6.3)
Group 3 Enterobacteriaceae	225 (4.8)	301 (5.5)	526 (5.1)
Nonfermenting Gram-negative bacilli	221 (4.7)	273 (5.0)	494 (4.8)
Enterococci	126 (2.7)	287 (5.2)	413 (4.0)
Other Gram-negative bacilli	63 (1.3)	71 (1.3)	134 (1.3)
Gram-negative cocci	22 (0.5)	110 (2.0)	132 (1.3)
Other Gram-positive cocci	23 (0.5)	44 (0.8)	67 (0.7)
*Vibrio cholerae*	58 (1.2)	0 (0.0)	58 (0.6)
Gram-positive bacilli	3 (0.1)	36 (0.7)	39 (0.4)
Anaerobic	15 (0.3)	23 (0.4)	38 (0.4)
Total	4,705 (100.0)	5,513 (100.0)	10,218 (100.0)

Enterobacteriaceae resistance groups: Group 1 = no beta − lactamase, Group 2 = low − level penicillinase, Group 3 = low − level cephalosporinase.

**Table 3:** Acquired bacterial resistance (AR) and relative change between 2010 and 2017 according to bacterial groups, Centre Pasteur du Cameroon, Yaoundé.

Bacterial group	Overall		2010		2017		Relative change	*p* value
	Isolates	AR (%)	Isolates	AR (%)	Isolates	AR (%)		
Enterobacteriacae	3,371	3,371 (100.0)	1,684	1,684 (100.0)	1,687	1,687 (100.0)	0.0	—
Nonfermenting GNB^∗^	392	380 (96.9)	167	162 (97.0)	225	218 (96.9)	-0.1	0.30
Other GNB	113	113 (100.0)	54	54 (100.0)	59	59 (100.0)	0.0	—
Staphylococci	419	330 (78.8)	266	183 (88.8)	153	147 (96.1)	+39.7	<0.001
Streptococci	505	391 (77.4)	300	195 (65.0)	205	196 (95.6)	+47.1	<0.001
Enterococci	408	408 (100.0)	126	126 (100.0)	282	282 (100.0)	0.0	—
Gram-negative cocci	124	123 (99.2)	15	15 (100.0)	109	108 (99.1)	-0.9	1
Anaerobic	17	17 (100.0)	9	9 (100.0)	8	8 (100.0)	0.0	—
*Vibrio cholerae*	56	56 (100.0)	56	56 (100.0)	0	0 (0.0)	—	1
Total	5,405	5,189 (96.0)	2,677	2,484 (92.8)	2,728	2,705 (99.2)	+6.9	<0.001

^∗^GNB: Gram-negative bacilli.

**Table 4:** Some specific resistances of clinical interest as per WHO priority resistances and their relative change between 2010 and 2017, Centre Pasteur du Cameroun, Yaounde.

Categories	Resistance features	Overall		2010		2017		Relative change (%)	*p* value
		Isolates	Feature frequency (%)	Isolates	Feature frequency (%)	Isolates	Feature frequency (%)		
WHO priority 1	Imipenem-R^1^*Acinetobacter baumannii*	167	15.6	65	6.2	102	21.6	+248.4	0.032
	Imipenem-R *Pseudomonas aerugino*sa	234	18.4	119	13.5	115	23.5	+74.1	< 0.01
	Imipenem-R Enterobacteriaceae	2,066	3.5	504	4.0	1,562	3.3	-17.5	< 10^−8^
	3GC^2^-R Enterobacteriaceae	3,643	32.1	1,818	23.8	1,825	40.4	+69.8	< 10^−15^

WHO priority 2	Vancomycin-R *Enterococcus spp*	412	0.5	126	0.0	284	0.7	—	1
	Methicillin-R *Staphylococcus aureus*	412	34.2	262	27.3	150	46.0	+68.6	< 10^−3^
	Fluoroquinolone-R *Campylobacter spp*	19	52.6	12	41.7	7	71.4	+71.4	0.3
	Fluoroquinolone-R *Salmonella spp*	144	5.6	103	3.9	41	9.5	+142.9	0.03
	3GC-R *Neisseria gonorrhoeae*	123	1.6	18	0.0	105	1.9	—	1
	Quinolone-R *Neisseria gonorrhoeae*	18	16.7	18	16.7	0	—	—	—
	Fluoroquinolone-R *Neisseria gonorrhoeae*	105	80.9	0	—	105	80.9	—	—

WHO priority 3	Penicillin nonsusceptible *S^3^. pneumoniae*	84	5.9	56	5.2	28	7.6	+47.0	0.09
	Ampicillin-R *Haemophilus* influenzae	62	70.9	9	55.6	53	73.6	+32.4	< 0.01
	Fluoroquinolone-R *Shigella spp*	128	0.8	53	0.0	75	2.7	—	1

Other specific resistances	Fluoroquinolone-R Enterobacteriaceae	3,447	45.1	1710	34.0	1737	56.0	+65.8	< 10^−15^
	3GC-R *Escherichia coli*	1,720	25.9	880	17.1	840	35.2	+105.8	< 10^−15^
	Fluoroquinolone-R *Escherichia coli*	1,693	49.9	867	37.4	826	61.6	+64.5	< 10^−15^
	Fluoroquinolone-R Mycoplasmas	3,505	63.8	1,636	62.8	1,869	64.6	+1.8	< 10^−3^
	Fluoroquinolone-I/R^4^ Mycoplasmas	3,505	97.6	1,636	98.6	1,869	96.8	-1.8	< 10^−3^
	Cyclin-R Mycoplasmas	3,505	27.2	1,636	32.7	1,869	22.3	-31.8	< 10^−13^
	Josamycin-R Mycoplasmas	3,505	1.10	1,636	1.1	1,869	1.2	+0.1	0.90

**Table 5:** Overall resistance pattern rates on five selected pathogens and their comparison between 2010 and 2017.

Resistance pattern	Overall		2010		2017		Relative change	*p* value
	Numbers	Rate, %	Numbers	Rate, %	Numbers	Rate, %		
Wild	5	0.1	2	0.1	3	0.1	29.5	1.00
Acquired resistance	787	17.6	393	18.1	394	17.1	-5.4	0.43
Multidrug resistance	3422	76.5	1628	74.9	1794	78.0	4.1	0.01
Extensively drug resistance	260	5.8	152	7.0	108	4.7	-48.8	<0.01
Total	4474	100.0	2175	100.0	2299	100.0	0.0	**—**

**Table 6:** Detailed resistance patterns per bacteria and their comparison between 2010 and 2017, Centre Pasteur du Cameroon, Yaoundé, Cameroon.

Bacteria	Resistance pattern	Numbers (%)			Relative change	*p* value
		Overall	2010	2017		
Enterobacteriaceae	AR	448 (13.2)	234 (13.7)	214 (12.7)	-7.3%	0.09
	MDR	2,748 (81.1)	1,362 (79.8)	1,386 (82.4)	+3.2%	
	XDR	193 (5.7)	110 (6.5)	83 (4.9)	-23.4%	
	Total	3,389 (100.0)	1,706 (100.0)	1,683 (100)	—	

*Pseudomonas aeruginosa*	AR	54 (27.7)	25 (25.9)	29 (29.9)	+15.5%	0.33
	MDR	109 (55.9)	53 (54.1)	56 (57.7)	+6.7%	
	XDR	32 (16.4)	20 (20.4)	12 (12.4)	-39.2%	
	Total	195 (100.0)	98 (100.0)	97 (100.0)	—	

*Acinetobacter spp*	AR	52 (34.7)	21 (37.5)	31 (33.0)	-10.7%	0.44
	MDR	84 (56.0)	32 (57.1)	52 (55.3)	-1.8%	
	XDR	14 (9.3)	3 (5.4)	11 (11.7)	+116.7%	
	Total	150 (100.0)	56 (100.0)	94 (100.0)	—	

*Enterococcus spp*	AR	78 (19.2)	11 (8.7)	67 (23.8)	+58.6%	< 10^−7^
	MDR	317 (77.9)	104 (82.6)	213 (75.8)	-8.1%	
	XDR	12 (2.9)	11 (8.7)	1 (0.4)	-95.4%	
	Total	407 (100.0)	126 (100.0)	281 (100.0)	—	

*Staphylococcus aureus*	Wild	5 (1.6)	2 (1.1)	3 (2.1)	+90.9%	<0.001
	AR	155 (46.6)	102 (54.0)	53 (36.8)	-17.2%	
	MDR	164 (49.2)	77 (40.7)	87 (60.4)	+48.4%	
	XDR	9 (2.7)	8 (4.2)	1 (0.7)	-83.3%	
	Total	333 (100.0)	189 (100.0)	144 (100.0)	—	

AR: acquired resistance; MDR: multidrug-resistant; XDR: extensively drug-resistant; PDR: pan drug-resistant.

## Data Availability

The authors of the present manuscript did not received special access privileges to the data which belong to the Centre Pasteur Cameroon, a public institution of the Ministry of public health. Most of them work at Centre Pasteur du Cameroun. To get access to the study data; a researcher should submit a request detailing the planned analysis on the requested data. Requests should be sent to the CPC (cpc@pasteur-yaounde.org) or to Dr. Tejiokem Mathurin (tejiokem@pasteur-yaounde.org). All requests will be reviewed by the scientific department.
